# Bone marrow mesenchymal stem cells combined with calcium alginate gel modified by hTGF-β1 for the construction of tissue-engineered cartilage in three-dimensional conditions

**DOI:** 10.3892/etm.2012.765

**Published:** 2012-10-25

**Authors:** SHAOBO ZHU, TAO ZHANG, CHEN SUN, AIXI YU, BAIWEN QI, HAO CHENG

**Affiliations:** Department of Micro-Orthopedics, Zhongnan Hospital, Wuhan University, Wuhan 430071, P.R. China

**Keywords:** gene transfection, chondrocytes, TGF-β1, TAZ, three-dimensional culture

## Abstract

The aim of this study was to investigate the feasibility of Ad-hTGF-β1-transfected bone marrow mesenchymal stem cells (BMMSCs) combined with calcium alginate gel for the construction of tissue-engineered cartilage under three-dimensional conditions. Rat BMMSCs were divided into three groups: the Ad-hTGF-β1 transfection group, the Ad-EGFP transfection group and the control group. The BMMSCs in the Ad-hTGF-β1 transfection group were continually grown. The compound of cell-calcium alginate gel was cultured in a constant temperature incubator. The morphology of cells was examined, and the proliferation of cells was detected by MTT assay. The results from real-time PCR showed that the average relative ratio of TGF-β1 and transcriptional coactivator with PDZ-binding motif (TAZ) in the Ad-hTGF-β1 group was comparable to that of the control group (P<0.05). Using western blotting and immunohistochemistry, strong expression of collagen II in the Ad-hTGF-β1 group was detected. The results from western blotting showed that the expression of TGF-β1 in the Ad-hTGF-β1 group was significantly increased compared with that of the other two groups. The differentiation of BMMSCs was induced by Ad-hTGF-β1 transfection into chondrocytes. TGF-β1 may promote the differentiation of BMMSCs into chondrocytes by TAZ. BMMSCs transfected by Ad-hTGF-β1 could be induced into chondrocytes. These three-dimensional conditions could preferably mimic cell growth patterns *in vivo*.

## Introduction

Seed cells and scaffold material are two main issues that are addressed in cartilage tissue engineering. Bone marrow mesenchymal stem cells (BMMSCs), being among the most commonly used seed cells in tissue engineering, have the ability to differentiate multi-directionally and can be generously amplified *in vitro*. Therefore, BMMSCs may be induced to differentiate into chondroblasts through various methods in order to construct cartilage-engineered tissue and provide seed cells.

BMMSCs *in vivo* mostly exist in the bone marrow at a static state, but when stimulated by physiological and pathological factors, their proliferation ability is demonstrated and BMMSCs differentiate into fat cells, bone cells and chondroblasts (1). Studies have shown that during the differentiation process from BMMSCs to chondrocytes, TGF-β1, one of the most important growth factors (2), can induce BMMSCs to differentiate into chondrocytes and promote the secretion of type II collagen and the synthesis and accumulation of extracellular matrix (3,4). Therefore, the TGF-β1 gene can be transferred into BMMSCs using gene transfer technology to ensure stable expression of TGF-β1, and BMMSCs can be persistently induced. This has become an ideal method in cartilage tissue engineering. Transcriptional enhancer factor TAZ (transcriptional coactivator with PDZ-binding motif) is one type of side line gene of the Yes-associated protein (YAP) that can regulate the transcription expression of Smad, BMP-2 and Runx, while the induction of TGF-β1 in BMMSCs is closely related to Smad, BMP-2, Runx and others (5,6). After transfection of TGF-β1, during the differentiation of BMMSCs to phenotypic chondrocytes, the method for effectively altering TAZ expression is still questionable. The present study was designed for a preliminarily investigation of this issue.

Most cell growth *in vivo* is wrapped in a three-dimensional environment similar as a niche box. For example, wrapped chondrocytes *in vivo* grow in cartilage matrix. At present, the antilinear of prepared and applicated porous scaffolds are usually much larger than that of cells; the cells are planted in such materials and can only spread and grow in an adherent manner, which is actually a two-dimensional (monolayer) culture model. This two-dimensional (monolayer) culture is not an effective manner to simulate cell growth *in vivo* in a three-dimensional micro-environment. Studies have shown that a three-dimensional environment is crucial for the maintenance of cell morphology and biological activity. Gel material has hydrophilicity which is suitable for cell embedding. It highly simulates the *in vivo* environment of cell growth, and provides a space similar to the natural substrate and chemical structure and signal transduction environment for cell growth (7). Alginate calcium is a type of saccharan composed of a different number of gulonic and mannuronic acids. Alginate calcium itself is biodegradable and biocompatible. This study was designed to investigate the feasibility of constructing tissue-engineered cartilage in a three-dimensional culture *in vitro* after embedding hTGF-β1 gene-transferred BMMSCs in alginate gel material.

## Materials and methods

### 

#### Experimental animals

Wistar rats, 120±15 g, male or female, were purchased from the Experimental Animal Center of Wuhan University (Wuhan, China).

#### Reagents

Fetal calf serum, L-DMEM medium containing 10% fetal calf serum, tryptase, rat anti-human TGF-β1 and collagen II polyclonal antibody were obtained. Goat anti-rat IgG secondary antibody, an immunohistochemistry kit, TRIzol reagent and a western blotting kit were obtained. The adenovirus with the EGFP gene (Ad-EGFP) and adenovirus with human transforming growth factor (Ad-hTGF)-β1 gene were constructed and preserved in our laboratory. Sodium alginate, calcium chloride and PCR primers were synthesized by Shanghai Biological Engineering Company.

#### Procurement and culture of BMMSCs

After ether anesthesia, the rats were sacrificed and soaked in 75% ethanol degeneration for 10 min. The femur was removed and the soft tissues were cleanly shaved. Both sides of the bone were opened with a rongeur, and the two femurs were placed in 10 ml L-DMEM medium containing 10% fetal calf serum. The bone marrow cavity was repeatedly flushed until turning white using medium in a 5-ml sterile syringe. The obtained cell suspension was repeatedly pipetted and mixed, and then the cell suspension was seeded in 60-mm sterile Petri dishes, and placed in a 95% humidified incubator at 37°C in 5% CO_2_ for incubation. The medium was replaced every 3–4 days, when the cells covered 70–80% of the dish. The cells were subsequently digested and subcultured with trypsin containing 0.25% EDTA. Third-generation cells were selected for use in the experiment.

#### Cell transfection and experimental groups

The third generation cells were seeded in 12-well plates at 1×10^5^/ml (400 *μ*l/well). When the cells reached 60% confluence, the cells were divided into three groups: the Ad-hTGF-β1-transfected group [serum-free medium with Ad-hTGF-β1 (MOI value of 200)], the Ad-EGFP-transfected group [serum-free medium with Ad-EGFP (MOI value of 200)]. The media in the two groups were replaced by complete medium after >48 h. The control group was cultured in complete medium with no treatment. The three groups of cells were placed in a 95% humidified incubator at 37°C in 5% CO_2_.

#### mRNA expression of TGF-β1 and TAZ in BMMSCs in the three groups as detected by real-time PCR

The various cultured cells were obtained 7 days after transfection, and total-RNA was extracted with TRIzol reagent according to the manufacturer’s instructions. β-actin was used as the housekeeping gene. Embedded dye SYBR-Green I was used for real-time quantitative PCR amplification. The primers were: TGF-β1 upstream primer, CGCAACAACGCAATCTATG and downstream primer, ACCAAGGTAACGCCAGGA, base length 204 bp. The PCR amplification system (25 *μ*l) contained 2.5 *μ*l Plus solution, 12.5 *μ*l SYBR-Green mix, 1 *μ*l of each of the upstream and downstream primer (5 pmol/*μ*l), 2.5 *μ*l cDNA and 5.5 *μ*l deionized water. Amplification conditions were: 50°C for 2 min, 95°C for 2 min, then a 95°C denaturation for 15 sec, and 58°C annealing for 15 sec, a 72°C extension for 45 sec, for a total of 40 cycles, then 72°C for 10 min.

#### Western blotting for the detection of TGF-β1 protein expression in the transfected BMMSCs

The cultured cells were obtained 7 days after transfection and were rinsed three times with cold PBS at 4°C. The lysis buffer-containing cocktail (1 ml lysis buffer plus 20 *μ*l cocktail, prepared 5 min prior to use) was added at 4°C and schizolysis was performed for 30 min. Percussion was carried out with a pipette 10 times every 5 min to collect the cell lysates. After centrifugation at 12,000 rpm for 10 min, the supernatant was collected at room temperature. Fifteen microliters was added/lane in an SDS-polyacrylamide gel electrophoresis tank, in Tris-glycine electrophoresis buffer for electrophoresis and was transferred to acetate film. The film was blocked with non-fat dry milk for 2 h in room temperature. Then blocking fluid and rat anti-human TGF-β1 (dilution 1:200) polyclonal antibody was added and oscillated for 2 h at 4°C and washed three times with PBS. Tris-Cl solution was added and incubated for 10 min at room temperature, then blocking solution and goat anti-rat IgG secondary antibody were added and incubated at room temperature for 1 h. The membrane was then transferred to Tris-Cl and incubated for 10 min. Horseradish peroxidase was added to produce the color images. Results were repeated three times, and GAPDH was used as a housekeeping gene.

### Preparation of the cell-calcium alginate gel compound

#### Preparation of the sodium polymannuronate suspension

The sodium alginate powder (Sigma) was dissolved in double distilled water at 12 g/l at room temperature agitated with a magnetic stirrer overnight. To fully dissolve the sodium alginate solution, the pH was adjusted to 7.0–8.0, and eas then reserved after high temperature and high pressure sterilization.

#### Preparation of compound cell-calcium alginate gel

The successfully transfected BMMSCs were digested, centrifugated and counted, and were separately and thoroughly mixed with sterile sodium alginate suspension at a final concentration of 1.0×10^7^ cells/ml. Excess calcium chloride was added at 102 mmol/l solution, and was kept standing for 20–30 min. Then the cells were washed 3–4 times with D-Hank’s solution, and the formative cell-calcium alginate gel compound was suspended in culture medium at 37°C in a 95% humidified incubator for culture. The culture medium was replaced every 3–4 days, and the cells were determined after 10 days of culture.

#### Proliferation of transfected cells determined by MTT assay and the number of BMMSCs in the calcium alginate gel material

The transfected cells in each group were obtained and digested with pancreatic enzyme containing 0.25% EDTA into a single-cell suspension, and the cell concentration was adjusted to 1×10^5^/ml and cells were seeded into a 96-well cell culture plate (200 *μ*l/well). At the same time point at 1, 2, 3, 4, 5, 6 days after transfection, the cells from three wells were randomly selected from the cells in each group, the culture medium was discarded, 20 *μ*l MTT was added (5 g/l) and culturing was carried out for 4 h. After removal of the supernatant, 150 *μ*l DMSO was added and oscillated for approximately 10 min. The extinction A value (OD) of each well was determined at 490 nm wavelength in a microplate reader, and the cell growth curve was drawn according to the measured values.

At the same time points 1, 2, 3, 4, 5, 6 days after cells were seeded in the alginate gel material, the cells were extracted and suspensed, and the cell concentration was adjusted to 1×10^5^/ml. The cells from the three wells were randomly selected from the cells in each group, the culture medium was discarded, 20 *μ*l MTT was added (5 g/l) and culturing was carried out for 4 h. After removing the supernatant, 150 *μ*l DMSO was added and oscillated for approximately 10 min. The extinction A value (OD) in each well was determined at 490 nm wavelength in the microplate reader and the cell growth curve was drawn according to the measured values.

#### Histological and histochemical staining

Ten days after the culture of the cell-calcium alginate gel compound *in vitro*, the compound was extracted and washed with PBS. The compound was fixed in 4% paraformaldehyde and embedded in paraffin according to the conventional method. It was cut into 4-*μ*m slices, and H&E, Masson’s and toluidine blue staining were performed, respectively, according to conventional methods, amd the results were examined under an inverted optical microscope.

#### Collagen II protein determined by immunohistochemistry

After the 10-day culture *in vitro*, the cell-calcium alginate gel compound was extracted and washed with PBS. The compound was fixed in 4% paraformaldehyde and embedded in paraffin according to a conventional method. It was cut into 4-*μ*m slices. SABC assay was used to detect collagen II expression. The primary antibody of collagen II was rat anti-human collagen II polyclonal antibody (1:100), and the secondary antibody was goat anti-rat IgG (1:200). The protocol was according to the manufacturer’s instructions, and the results were observed and tested under an inverted optical microscope and photographed.

#### Data analysis

Data are expressed as the means ± SD, said the group. The comparison of group differences was carried out using the analysis of variance. SPSS13.0 statistical software was used for statistical analysis, and P<0.05 indicated a significant difference.

## Results

### 

#### Morphology of BMMSC in primary culture and cells cultured in calcium alginate gel

**Figure 1:**
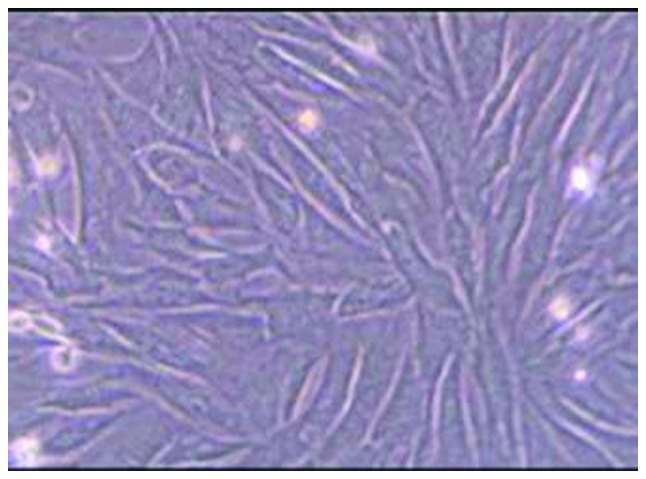
Morphology of the second generation BMMSCs cultured for 2 days. BMMSCs exhibit adherence, undergo colony formation and are distributed uniformly. The cells appear spindle-shaped.

**Figure 2:**
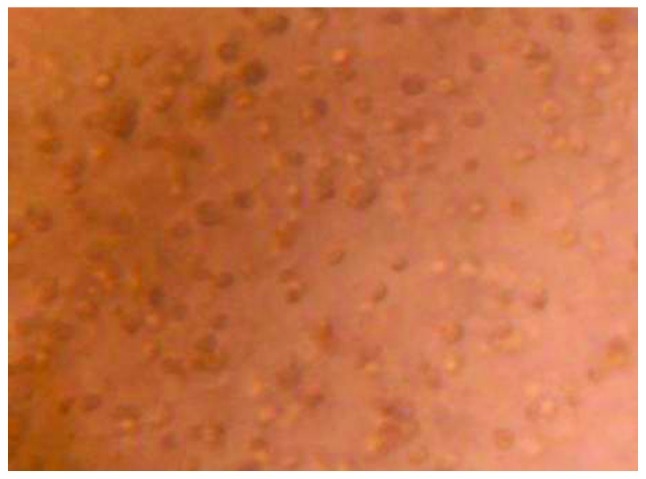
Morphology of the BMMSCs in calcium alginate gel. BMMSCs are evenly distributed in the gel layer, and their shape is spherical.

After 72 h of primary culture, BMMSCs were observed to exhibit adherence and colony formation. The cells were spindle-shaped and polygonal-based which continued for the 7- to 9-day culture. When the cells reached 80–90% confluence, the speed of cell proliferation was significantly rapid after passage. The cells were uniform and continued to grow. After 2–3 days, the cells reached 80–90% confluence, and the cells exhibited a long spindle-shape (Fig. 1). After BMMSCs were grown in calcium alginate gel, they were evenly distributed in the gel layer, and their shape was spherical (Fig. 2).

#### mRNA expression of TAZ and TGF-β1 in the transfected cells as determined by real-time PCR

**Figure 3:**
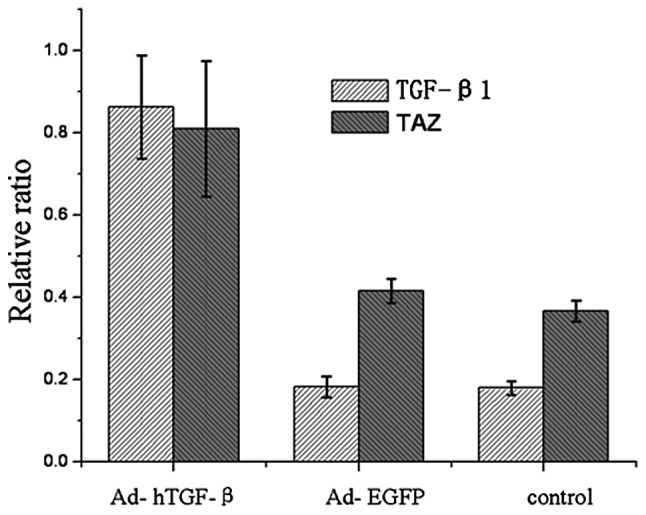
Real-time PCR results showed that TGF-β1 and TAZ expression in the Ad-hTGF-β1-transfected group was significantly higher than levels in the Ad-EGFP-transfected and control groups.

As shown in Fig. 3, 7 days after transfection, the mRNA expression of TGF-β1 and TAZ in the Ad-hTGF-β1-transfected group was significantly higher than levels in the Ad-EGFP-transfected and the control groups.

#### Protein expression of TGF-β1 in the transfected BMMSCs as determined by western blotting

**Figure 4:**
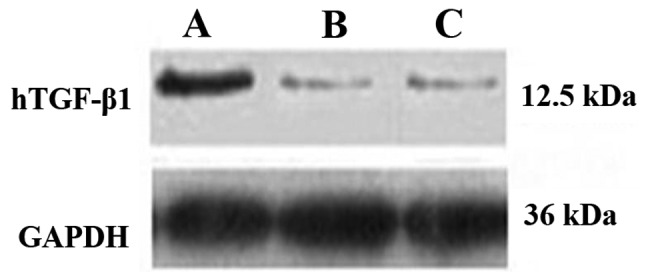
TGF-β1 and collagen II expression in the (A) Ad-hTGF-β1-transfected group, (B) Ad-EGFP-transfected group and (C) control group as determined by western blotting.

As shown in Fig. 4, 7 days after transfection, the protein expression of TGF-β1 in the Ad-hTGF-β1-transfected group was significantly higher than that of the Ad-EGFP-transfected and control groups (Fig. 4).

#### Cell proliferation ability in the transfected BMMSCs as determined by MTT assay

**Figure 5:**
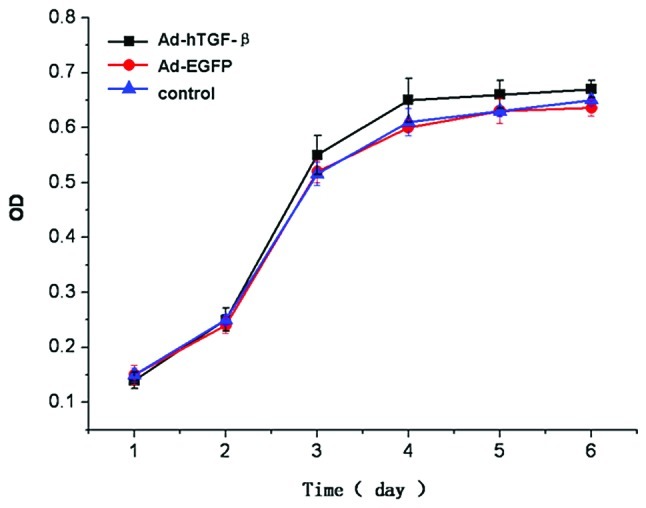
Cell growth curves of the three experimental cell groups had similar shapes and the OD values at each time point were not significantly different.

**Figure 6:**
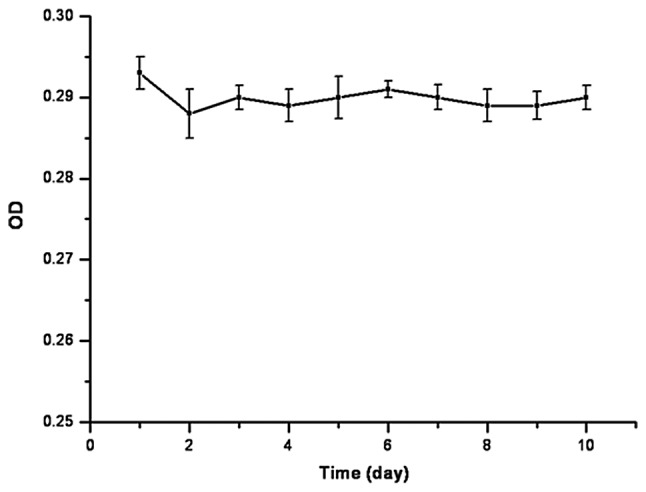
According to i*n vitro* time, the number of cells were relatively stable, and the OD values at each time point were not significantly different.

The results from the MTT colorimetric assay showed that after transfection, days 1 and 2 after cell seeding in the three groups was considered to be a period of delitescence, day 3 was considered as the logarithmic growth phase, day 4 was considered as the platform phase. The cell growth curves for the three groups were similar, and the difference in absorbance (OD) at each time point was not statistically significant (P=0.718, 0.670, 0.246, 0.192, 0.172, 0.094) (Fig. 5). At the same time, after the cells were seeded on the calcium alginate gel, the cell growth curve did not change significantly, and difference in the absorbance (OD) at each time point was not statistically significant (P=0.473) (Fig. 6)

#### Histological and histochemical observations

**Figure 7:**
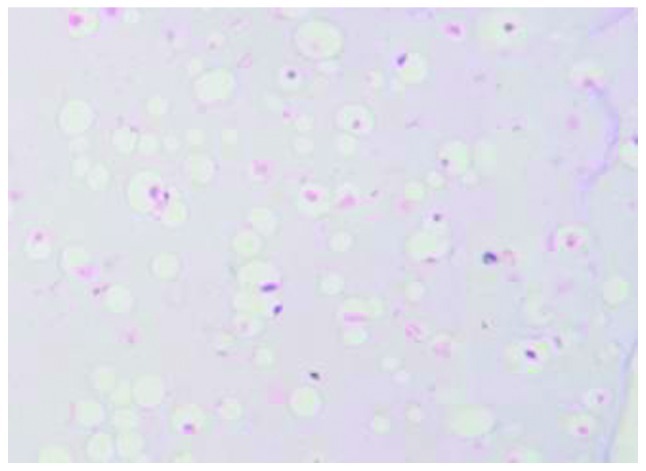
H&E staining of the calcium alginate gel. A large number of cartilage lacunaes were formed in the gel material.

**Figure 8:**
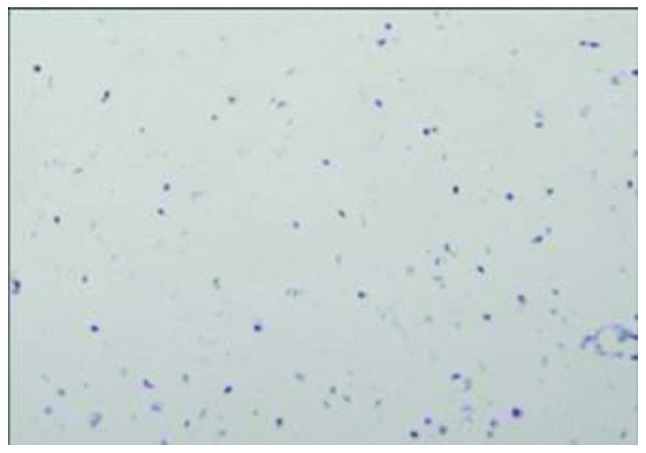
Masson’s staining in the calcium alginate gel. Masson’s staining showed the synthesis and secretion of type II collagen in the gel material.

**Figure 9:**
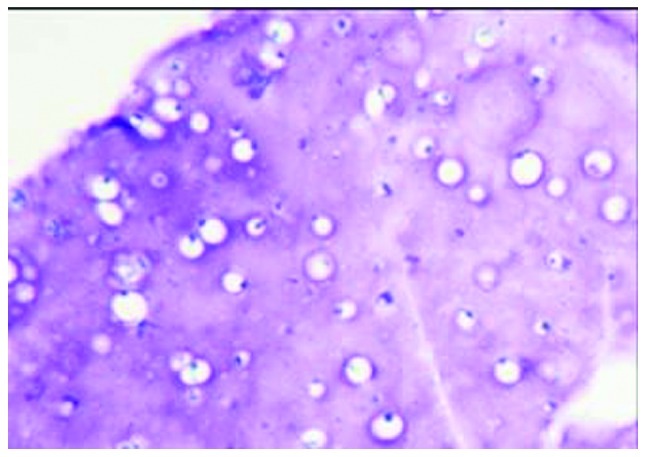
Toluidine blue staining in the calcium alginate gel. Toluidine blue staining confirmed the synthesis and secretion of proteoglycan in gel material.

H&E staining showed that a large number of cartilage lacunae were formed in the gel material, and the nucleolus was clearly visible (Fig. 7). Masson staining showed the synthesis and secretion of type II collagen in the gel material (Fig. 8). Toluidine blue staining confirmed the synthesis and secretion of proteoglycan in the gel material (Fig. 9).

#### Type II collagen determined by immunohistochemistry

**Figure 10:**
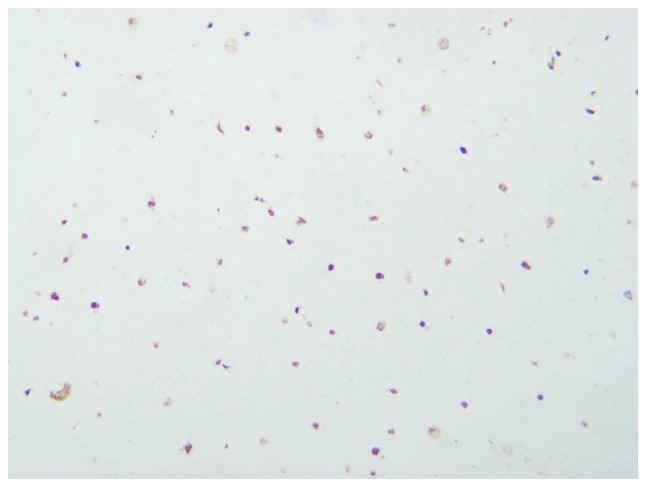
Immunohistochemistrial staining of type II collagen in the calcium alginate gel. Brown particles were evident in the gel material, and type II collagen was positively expressed in the calcium alginate gel.

The results from type II collagen determined by immunohistochemistry showed that 10 days of culture *in vitro* after the Ad-hTGF-β1-transfected cells were seeded in the gel material, brown particles were present in the gel material. This indicated that BMMSCs synthesized and secreted type II collagen (Fig. 10).

## Discussion

Seed cells for application in the tissue engineering of cartilage include chondrocytes and mesenchymal stem cells, yet autologous chondrocytes are not easily obtained, the source is limited and their use easily leads to cartilage damage of draw material parts. Previous studies have shown that only 1.8–4.5×10^5^ chondrocytes can be extracted from a trauma patient with knee injury with a single femoral condyle articular cartilage (2), which is not enough for chondrocyte transplantation (3). In contrast, mesenchymal stem cells are easily obtained, can be amplified *in vitro* and have a strong ability to differentiate into cartilage cells, particularly mesenchymal stem cells. Thus, we chose bone marrow mesenchymal stem cells as seed cells in this study. TGF-β1 is known to be a growth factor in bone that can induce BMMSCs to differentiate into chondrocytes, TGF-β1 is also one of the most important factors causing BMMSCs to differentiate into cartilage (4). In view of these facts, gene transfer technology was used to transfer the TGF-β1 gene into BMMSCs using a standard method. TGF-β1 can steadily induce BMMSC differentiation but does not affect the proliferation rate of BMMSCs. In this study, after BMMSCs were transfected with Ad-hTGF-β1, the results from real-time PCR and western blotting showed that TGF-β1 expression in the Ad-hTGF-β1-transfected cells was significantly higher than levels in the Ad-EGFP-transfected and control group cells; a fact indicating that we successfully constructed BMMSCs with high expression of TGF-β1.

The role of TGF-β1 in the differentiation of BMMSCs was found to be affected by Smad, BMP-2 and Runx genes (5). TAZ is one of the YAP sideline genes, and regulates the expression of Smad, BMP-2 and Runx at the transcription level, and it also affects the life of BMMSCs (6). In this study, we designed primer sequences to detect TAZ mRNA, and the expression differences of TAZ mRNA were detected between the experimental and control groups through real-time quantitative PCR. The results showed that the level of TAZ mRNA in the Ad-hTGF-β1-transfected cell group was significantly higher than levels in the Ad-EGFP-transfected and control groups (P<0.05). This indicates that BMMSCs transfected by Ad-hTGF-β1 induced BMMSC differentiation into chondrocytes due to the increased expression of TGF-β1, while the mRNA level of TAZ was also increased in this process. Therefore, we speculated that TAZ may play a regulatory role in the process of TGF-β1-induced BMMSC differentiation to chondrocyte phenotype. However, several studies have also demonstrated that in the process of TGF-β1-induced BMMSC differentiation into osteoblasts, the expression level of TAZ is increased (7,8). Therefore, it remains to be investigated how TAZ regulates the process of TGF-β1-induced BMMSC differentiation to chondrocytes and osteoblasts.

In a healthy organism, in addition to blood cells in the circulatory system, other cells are grown in a relatively stable three-dimensional space structure. In this structure, the cells are packaged in the extracellular matrix, and soluble growth factors distributed in the extracellular matrix are able to bind to receptors on the cell surface and play a role in regulating the activity of cell biology (9). At the same time, interactions between cells also play a regulatory role in the biological activity of cells. For mesenchymal stem cells, the extracellular matrix and cell interactions also play an important role in maintaining the morphology of mesenchymal stem cells, a fact that directly affects the proliferation and differentiation activity of mesenchymal stem cells themselves (10,11). These features require us to take into account the *in vivo* environment of the mesenchymal stem cells in the study of tissue engineering, and thus we tried to simulate the three-dimensional spatial structure for the growth of cells in order to maintain the biological activity of mesenchymal stem cells when constructing tissue-engineering scaffolds.

Research has revealed that a two-dimensional (single layer) condition is not a good method for maintaining the biological and biochemical properties of cells (1), particularly those of mesenchymal stem cells. If the cells are placed in a single- or two-dimensional culture environment, the ability of cells to differentiate into cartilage is difficult to maintain. Therefore, material chemistry, structure, processing technology and bioauxology can be used to design a three-dimensional cell culture matrix for tissue engineering with an adequate geometric and chemical composition similar to the natural extracellular matrix signaling system. In addition, this unique structure is conducive to the transmission of soluble molecules, as well as the exchange of nutrients and cell metabolism waste (12). The traditional material for cartilage tissue engineering mainly includes polyvinyl alcohol, polyglycolic acid, polylactic acid, chitosan and a mixture according to different proportions. The porous structure of the scaffolds is ensured by a freeze-drying method, and the seed cells are then seeded in this scaffold. Concerning the porous scaffold prepared using this method, the pore size is generally between 200–400 *μ*m (13), while the diameter of the cell is only a few microns; much smaller than the pore size of the scaffold. The cells seeded to grow in material with such pores inevitably make the cells spread and attach to the surface of the material in an adherent manner, which is actually a two-dimensional (single) model. It is difficult to maintain the spherical shape of cells, and the latter is an indispensable condition for mesenchymal stem cells exerting their biological properties (14). In contrast, gel-like material is prepared according to the physical and chemical properties of the biological material itself rather than by a freeze-drying method. The pore of this material is very small, while a large number of charged groups are on the surface of gel, which makes the material highly hydrophilic. This precisely simulates the body’s natural growth environment of cartilage cells (15). This is extremely beneficial for the differentiation of mesenchymal stem cells to cartilage cells.

Alginate (e.g. sodium alginate), which is a type of anionic polysaccharide without a side chain derived from brown algae, exists as a solid when it interacts with polyvalent cations (e.g. calcium) (1,16). Alginate displays outstanding biocompatibility with the host and seed cells in condition of maintaining their own invariant characteristics (1). In animal experiments, gel complexes with cartilage cells and alginate has been successfully used in cartilage transplantation (17,18). Alginate calcium arises from the crosslink of sodium alginate and calcium chloride. It has superior biocompatible and absorptive abilities, and has a porous structure that contributes to material exchange required by the metabolism (19). In addition, the hydrophilic ability of alginate makes cells difficult to attach to the surface of alginate material, a fact that helps to maintain the spherical structure and stabilize the differentiation of cells (2). A number of studies have shown that alginate can be used as a three-dimensional material to culture chondrocytes, since alginate material can simulate the environment of articular cartilage and induce cartilage cells to secrete proteoglycans and maintain the surrounding chondrocytes (20). However, it is still unclear whether BMMSCs gene-modified using an alginate calcium-gel complex can be used to successfully construct tissue engineered cartilage *in vitro*. In the present study, after BMMSCs were successfully transfected by Ad-hTGF-β1, they were generously amplified *in vitro*. The cells at 1.0×10^7^/ml were mixed in sodium alginate (17) to become a cell suspension, and calcium chloride was added and fully cross-linked with the sodium alginate-cell suspension and finally made into a hydrogel combined with calcium alginate-cells. BMMSCs were observed in the gel material in a uniformly distributed manner under an inverted microscope and grew in a spherical state (Fig. 2), which is required to maintain the stability of the cell morphology, and simulate the grow morphous of cells in the body to ensure the biological and biochemical characteristics of cells. It effectively avoids the shortcoming of adherent growth of cells cultured in a common two-dimensional (single) manner. We used MTT assay *in vitro*, to detect the cell number and found that the number of cells in the alginate gel at different times was not significantly different (P=0.473). This suggests that the cells embedded in the alginate gel can obtain the required nutrients from the medium *in vitro* to ensure the required energy of normal cells and maintain the cell activity, yet at the same time, the cells do not undergo rapid proliferation in the gel material as the gel material is unable to provide adequate space for cell proliferation.

To confirm the differentiation of cartilage cells in gel using BMMSCs modified by hTGF-β1 combined with alginate calcium, after 10 days of culture *in vitro*, we adopted H&E, toluidine blue and Masson’s staining for application to the alginate-cells combined with gel materials. The results showed that a large number of lacunae and a great amount of cartilage proteoglycans and collagen was found in the gel material. The results from immunohistochemistry showed that type II collagen can be produced in the cultured cells from gel material. This indicates that after the Ad-TGF-β1-transfected mesenchymal stem cells were cultured in alginate calcium gel they were steadily differentiated into cartilage.

Our preliminarily experimental results showed that BMMSCs modified by the hTGF-β1 gene can be used as seed cells for the tissue engineering of cartilage, while calcium alginate gel material provides the required three-dimensional structure for cartilage differentiation of seed cells. Alginate gel can be used as a suitable carrier material for cartilage tissue engineering. However, a key defect of gel material is that the mechanical properties cannot reach the requirements of vito-dynamics. We expect that in future studies, three-dimensional alginate gel materials will be able to be combined with the superior mechanical ability of a porous scaffold to develop more comprehensive tissue engineering scaffolds. In addition, the detailed mechanism involved in the three-dimensional culture environment affecting stem cell proliferation and differentiation remains to be further investigated.
